# Prognostic Values of Long Noncoding RNA GAS5 in Various Carcinomas: An Updated Systematic Review and Meta-Analysis

**DOI:** 10.3389/fphys.2017.00814

**Published:** 2017-11-02

**Authors:** Qunjun Gao, Haibiao Xie, Hengji Zhan, Jianfa Li, Yuchen Liu, Weiren Huang

**Affiliations:** ^1^Key Laboratory of Medical Reprogramming Technology, Shenzhen Second People's Hospital, Graduate School of Guangzhou Medical University, Shenzhen, China; ^2^Guangzhou Medical University, Guangzhou, China; ^3^Shantou University Medical College, Shantou, China; ^4^Shenzhen University, Shenzhen, China

**Keywords:** lncRNA, GAS5, cancer, prognosis, lymph node metastasis, high tumor stage, meta-analysis

## Abstract

The growth arrest-specific transcript 5 (GAS5) is a long noncoding RNA with low expression in multiple cancers. This meta-analysis aims to explore the association between GAS5 expression levels and cancer patients' prognosis. We collected all the relevant literatures about GAS5 expression levels associated with overall survival (OS), lymph node metastasis (LNM) and high tumor stage (II/III/IV) (HTS) from the PubMed and Web of Science. The hazard ratio (HR) and the corresponding 95% confidence interval (CI) were calculated to evaluate the link strength between GAS5 and cancer prognosis. A total of 934 patients from 14 studies were included to the present meta-analysis, according to the inclusion and exclusion criteria. The results demonstrated that low expression of GAS5 could predict poor OS in cancer patients (HR = 1.955, 95% CI: 1.551–2.465, *P* < 0.001). Meanwhile we also analyzed the following cancers independently: hepatocellular carcinoma (HR = 1.893, 95% CI: 1.103–3.249, *P* = 0.021) and urothelial carcinoma (HR = 1.653, 95% CI: 1.185–2.306, *P* = 0.003). Compared to the high GAS5 expression group, additionally, patients with low GAS5 expression in tumor tissues were more prone to lymph node metastasis (OR = 0.234, 95%CI: 0.153–0.358, *P* < 0.001) and high tumor stage (OR = 0.185, 95% CI:0.102–0.333, *P* < 0.001). In conclusion, this meta-analysis showed that GAS5 might be served as a novel biomarker for predicting prognosis in various types of cancers.

## Introduction

Cancer has become a global health problem. In recent years, the incidence of cancer has been increased year by year. According to WHO estimates, 14.1 million new cancer patients and 8.2 million deaths from cancer occurred worldwide in 2012 and more than 20 million new cases of cancer will be expected as early as 2025 (Ferlay et al., 2015). At present, cancer treatment includes surgery, radiotherapy, chemotherapy andetc., but the 5 years survival rate is still not ideal, especially some patients with lymph node metastasis or high stage tumor (Saika and Sobue, 2013). Therefore, it is important to find a new biological target that plays a guiding role in the carcinogenesis to detect cancer. It is also more conducive to early detection, early diagnosis and early treatment of tumor patients.

Long non-coding RNAs (lncRNAs) are noncoding RNAs with a length of more than 200 nucleotides that regulate gene expression (Mattick and Makunin, 2006). They were described as “noise,” and did not attract much attention in the past few decades (Ponjavic et al., 2007). With the application of whole genome sequencing and microarray, lncRNAs have attracted more and more attentions (Batista and Chang, 2013; Tang et al., 2013). The increasing evidence show that lncRNAs play a pivotal role in the development and progression of tumors, which means that they can be used as biomarkers for some tumors (Fang et al., 2017; Liu et al., 2017; Sun et al., 2017; Yang et al., 2017). However, only a few number of lncRNAs have corresponding functional features, and most of the functions of lncRNAs remain unclear.

The growth arrest-specific transcript 5 (GAS5) is a rising star among tumor-suppressive lncRNAs among all the kinds of lncRNAs (Ma et al., 2016). Recent studies have shown that GAS5 plays a key role in a variety of human diseases and participates a variety of biological processes, such as cell proliferation, cell apoptosis, epithelial-mesenchymal transition and etc. (Tan et al., 2017; Tao et al., 2017; Wen et al., 2017; Yang et al., 2017). Meanwhile, GAS5 is also involved in the progression of many types of cancer, such as bladder cancer (BC) (Zhang et al., 2017), colorectal cancer (CRC) (Yin et al., 2014; Li et al., 2017), non-small cell lung cancer (NSCLC) (Shi et al., 2015; Wu et al., 2016), breast cancer (BRC) (Li W. et al., 2016), hepatocellular carcinoma (HCC) (Tu et al., 2014; Chang et al., 2016; Hu et al., 2016), epithelial ovarian cancer (EOC) (Gao et al., 2015), gastric cancer (GC) (Sun et al., 2014), cervical cancer (CEC) (Cao et al., 2014), and head and neck squamous cell carcinoma (HNSCC) (Gee et al., 2011). The clinic pathological features of the patients, such as overall survival (OS), lymph node metastasis (LNM) and high tumor stage (II/III/IV) (HTS), are also highly correlated with the level of GAS5 expression in these cancers (Gee et al., 2011; Cao et al., 2014; Sun et al., 2014; Tu et al., 2014; Yin et al., 2014; Gao et al., 2015; Shi et al., 2015; Chang et al., 2016; Hu et al., 2016; Li J. et al., 2016; Wu et al., 2016; Droop et al., 2017; Li et al., 2017; Zhang et al., 2017). All these indicate that GAS5 can be a novel prognostic biomarker in unique cancer. To shed light on the relationship between GAS5 and cancer prognosis, the meta-analysis on the association between the expression of GAS5 and the prognosis of cancer is required. Although a meta-analysis has reported that the expression of GAS5 predicts poorer survival outcomes, only 4 literatures have been included in that work and the results may be incidental (Song et al., 2016). To verify the accuracy of the previous results, the present meta-analysis with 14 studies may provide a more accurate conclusion.

## Materials and methods

### Literature collection

We searched potentially eligible literatures through PubMed, Web of Science to locate articles (published during March 2011 to April 2017), including articles referenced in the publications. We used “GAS5 or growth arrest specific 5” AND “cancer or tumor or carcinomas or neoplasm” as the keywords, in order to identify potentially relevant studies. Citation lists of retrieved articles were searched manually to ensure sensitivity of the search strategy.

### Inclusion and exclusion criteria

All the eligible study data elements were independently assessed and extracted by two investigators. For inclusion in this meta-analysis, the studies met the following criteria: the association between GAS5 and cancer prognosis (OS) was investigated; patients were grouped according to the expression levels of GAS5;related clinic pathologic parameters were described, such as LNM, TNM and sufficient original data for calculating a hazard ratio (HR) with its 95% confidence interval (CI). Exclusion criteria are as the following: Duplicate publications; irrelevant to cancer, GAS5, or cancer prognosis; animal studies, letters, editorials, expert opinions, abstracts, case reports and reviews; studies without usable data.

### Data extraction

According to the inclusion and exclusion criteria, two investigators extracted and reviewed the data independently (GQJ, XHB), and disagreements were discussed with two investigators (ZHJ, LYC) in conference. The following data were extracted: first author, publication date, country of origin, tumor type, total number of patients, number of high GAS5 expression group and low GAS5 expression group, number of patients with LNM, number of patients with HTS, detection method of GAS5 expression levels, follow-up month and cut-off values, multivariate analysis, hazard ratios (HRs), and corresponding 95% CI for OS.

### Statistical methods

Meta-analysis was performed using Stata12.0 software. Pooled hazard ratios (HRs) were extracted from the included studies; the log HR and standard error (SE) were used for aggregation of the survival results (Tierney et al., 2007). To determine the heterogeneity among the included studies, chi-square-based Q test and I^2^ statistics were used (Higgins et al., 2003). If the *P* < 0.1 or I^2^ > 50%, it means that significant heterogeneity existed among the included studies, thus the random-effects model was adopted to analyze the results. The fixed-effects model was applied when between-study heterogeneity was absent (*P* > 0.1 and I^2^ < 50%). The potential publication bias was assessed using the Eegg'stest and *P* < 0.05 was considered representative of statistically significant publication bias. Sensitivity analysis was performed by sequential omission of each individual study in order to validate the stability of outcomes in the present meta-analysis.

### Quality assessment of primary studies

Two investigators (GQJ, XHB) performed the quality assessment of primary studies independently. We evaluated all eligible studies' quality by using the Newcastle-Ottawa Scale (NOS) for assessing the quality of studies in meta-analyses (Zeng et al., 2015). The higher scores indicated better methodological quality.

## Results

### Characteristics and eligible studies

**Figure 1:**
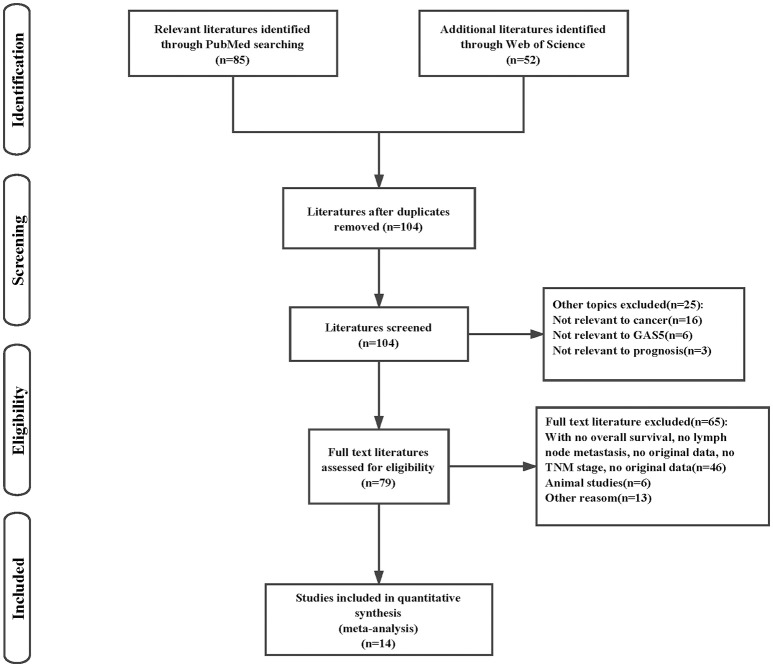
Flow chart presenting the steps of literature search and selection.

The initial search of the electronic database retrieved 137 literatures. After removing the duplicates, 104 articles were remained. Then we carefully screened the title and abstract, 25 literatures were excluded because the studies were irrelevant. Upon further review of the full articles, the articles with no survival outcomes, lymph node metastasis, TNM stage, animal testing and other factors were excluded. 14 articles were eventually selected for the present meta-analysis (Figure 1). A total of 934 patients were included among these studies, with a maximum sample size of 106 and a minimum sample size of 24 patients (Mean 67). The publication years of the included studies were between 2011 and 2017. In these studies, one was from UK, one was from Germany and 8 were from China. A total of 9 different types of cancer were evaluated in studies of this meta-analysis (3 hepatocellular carcinoma, 2 colorectal cancer, 2 non-small cell lung cancer, 2 urothelial carcinoma, 1 breast cancer, 1 epithelial ovarian cancer, 1 gastric cancer, 1 cervical cancer and 1 head and neck squamous cell carcinoma). The expression of GAS5 was detected by qRT-PCR and the cut-off values included in the studies were inconsistent. All diagnoses of LNM and TNM were based on pathology. Hazard ratios with the corresponding 95% CIs were extracted from the graphical survival plots and the articles. The main characteristics of the eligible studies were summarized in Table 1. The Newcastle-Ottawa Scale (NOS) confirmed that all the studies were of high quality (Table 2).

**Table 1:** Characteristics of included studies in this meta-analysis.

**Study**	**Year**	**Country**	**Cancer type**	**Total number**	**Detection method**	**Cut-off**	**GAS5 expression**						**Survival analysis**	**Multivariate analysis**	**HR Statistic**	**Hazard Ratios(L/H) (95%CI)**	**Follow-up months**
							**High**	**High with HTS**	**High with LNM**	**Low**	**High with HTS**	**Low with LNM**					
Zhang	2016	China	BC	82	qRT-PCR	Median	41	NA	NA	41	NA	NA	OS	Yes	Rep	2.073 (1.231–3.490)	60 (Total)
Li	2017	China	CRC	24	qRT-PCR	Median	12	NA	3	12	NA	9	NA	NA	NA	NA	NA
Droop	2017	Germany	UC	106	qRT-PCR	Median	53	NA	NA	53	NA	NA	OS	Yes	Rep	1.414 (0.917–2.179)	NA
Wu	2016	China	NSCLC	48	qRT-PCR	X-tile algorithm	9	3	2	39	27	25	NA	NA	NA	NA	NA
Li	2016	China	BRC	86	qRT-PCR	X-tile algorithm	15	NA	NA	71	NA	NA	OS	Yes	SC	0.65 (0.08–5.47)	60 (Total)
Hu	2015	China	HCC	32	qRT-PCR	X-tile algorithm	11	NA	NA	21	NA	NA	OS	Yes	SC	2.08 (0.73–5.92)	30 (Total)
Chang	2015	China	HCC	50	qRT-PCR	Mean	25	NA	NA	25	NA	NA	OS	Yes	SC	1.96 (0.96–4.00)	60 (Total)
Shi	2015	China	NSCLC	72	qRT-PCR	X-tile algorithm	26	13	11	46	36	22	NA	NA	NA	NA	NA
Gao	2015	China	EOC	60	qRT-PCR	X-tile algorithm	29	16	12	31	29	28	NA	NA	NA	NA	NA
Yin	2014	China	CRC	66	qRT-PCR	Mean	33	24	14	33	32	20	OS	Yes	SC	2.31 (0.51–10.45)	60 (Total)
Tu	2014	China	HCC	71	qRT-PCR	Mean	20	NA	4	51	NA	28	OS	Yes	SC	1.43 (0.37–5.49)	60 (Total)
Sun	2014	China	GC	89	qRT-PCR	Median	45	29	NA	44	39	NA	OS	Yes	Rep	2.46 (1.42–4.26)	40 (Total)
Cao	2014	China	CEC	102	qRT-PCR	Median	58	NA	12	44	NA	32	OS	Yes	Rep	3.217 (1.684–6.964)	44 (Mean)
Gee	2011	UK	HNSCC	46	qRT-PCR	Median	23	NA	NA	23	NA	NA	OS	NA	SC	2.40 (0.31–18.72)	60 (Total)

*BC, bladder cancer; CRC, colorectal cancer; UC, urothelial carcinoma; NSCLC, non-small cell lung cancer; BRC, breast cancer; HCC, hepatocellular carcinoma; EOC, epithelial ovarian cancer; GC, gastric cancer; CEC, cervical cancer; HNSCC, head and neck squamous cell carcinoma; UK, United Kingdom of Great Britain and Northern Ireland; HTS, high tumor stage(II/III/IV); LNM, lymph node metastasis; DM, distant metastasis; qRT-PCR, quantitative real-time polymerase chain reaction; OS, overall survival; NA, not available; Rep, reported; SC, survival curve; L/H, low expression of GAS5/high expression of GAS5*.

**Table 2:** Quality assessment of eligible studies (Newcastle-Ottawa Scale).

**Study**	**Selection**			**Comparability**			**Outcome**		**Total**
	**Adequacy of case definition**	**Number of case**	**Representativeness of the cases**	**Ascertainment of exposure**	**Ascertainment of detection method**	**Ascertainment of cut-off**	**Assessment of outcome**	**Adequate follow up**	
Zhang 2016	1	1	1	1	1	1	1	1	8
Li 2017	1	0	1	1	1	1	1	0	6
Droop 2017	1	1	1	1	1	1	1	0	7
Wu 2016	1	0	1	1	1	1	1	0	6
Li 2016	1	1	1	1	1	1	1	1	8
Hu 2015	1	0	1	1	1	1	1	0	6
Chang 2015	1	1	1	1	1	1	1	1	8
Shi 2015	1	1	1	1	1	1	1	0	7
Gao2015	1	1	1	1	1	1	1	0	7
Yin 2014	1	1	1	1	1	1	1	1	8
Tu 2014	1	1	1	1	1	1	1	1	8
Sun 2014	1	1	1	1	1	1	1	1	8
Gao 2014	1	1	1	1	1	1	1	1	8
Gee 2011	1	0	1	1	1	1	1	1	7

### Meta-analysis result

#### Association between GAS5 and OS in seven types of cancers

**Figure 2:**
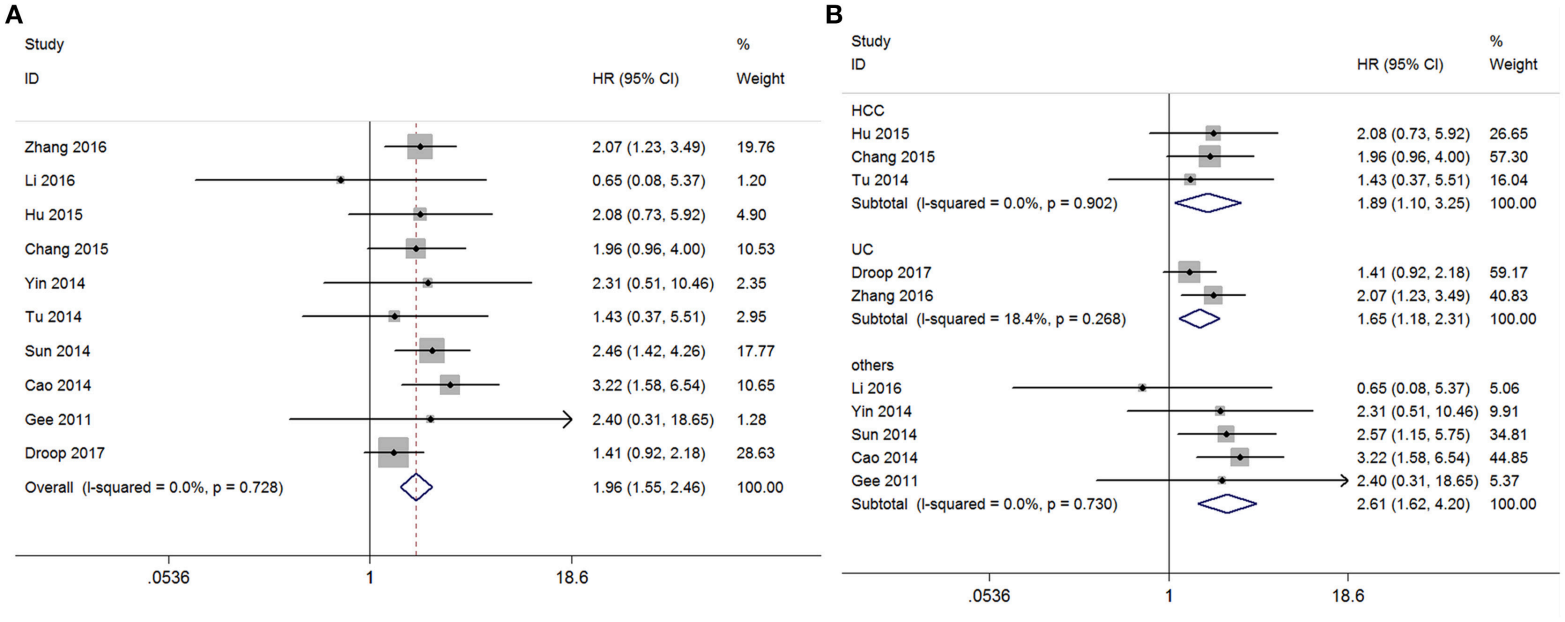
Meta-analysis of the pooled HRs of OS of different types of cancer with the level of GAS5 expression. **(A)** Forest plot for the correlation between GAS5 expression levels and OS in different cancer patients. **(B)** Subgroup analysis of HRs of OS by factor of different types of cancer.

Among the included studies, 10 reported the overall survival (OS) of 730 patients according to GAS5 expression levels. In order to study the relationship between GAS5 expression level and prognosis, the fixed-effect model was used to calculate the pooled HR with corresponding 95% CI because heterogeneity analysis showed that low between-study heterogeneity among those nine studies for GAS5 expression was found (I^2^ = 0.0%, P(H) = 0.728). We found an inverse relationship that low expression of GAS5 might be associated with poor overall survival outcome (*HR* = 1.955, 95% CI:1.551–2.465, *P* < 0.001, fixed-effect model) (Figure 2). In a subgroup analysis of cancer sites, we also found the similar significant adverse association between levels of GAS5 and OS in the following cancers (low/high): HCC (HR = 1.893, 95% CI: 1.103–3.249, *P* = 0.021, P(H) = 0.902), UC (HR = 1.653, 95% CI: 1.185–2.306, *P* = 0.003, P(H) = 0.268) and HR for the subgroup of other cancers was 2.641 (95%CI: 1.625–4.204, *P* < 0.001, P(H) = 0.730). We didn't perform subgroup analyses for CRC, BRC, EOC, GC, CEC, and HNSCC, because there is only one paper investigating these associations between GAS5 and OS (Figure 2) in each cancer type. Compared with the high expression group, the low GAS5 expression group indicates a poorer OS which was confirmed statistically significant.

#### Association between GAS5 and LNM

**Figure 3:**
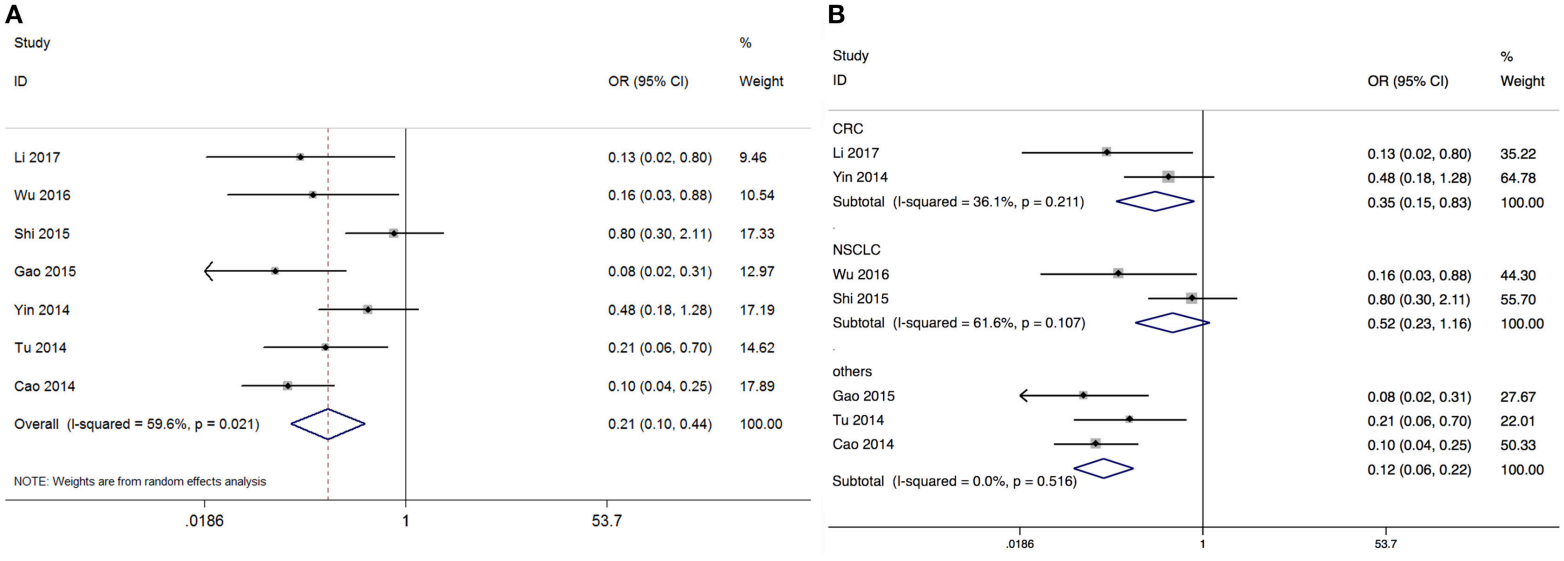
Meta-analysis of the LNM of different types of cancer with the level of GAS5 expression. **(A)** Forest plot for the correlation between GAS5 expression levels and LNM in different cancer patients. **(B)** Subgroup analysis of lymph node metastasis by factor of different types of cancer.

Based on the differential expression levels of GAS5, seven studies reported 443 patients with lymph node metastasis. Because of the significant between-study heterogeneity (I^2^ = 59.6%, *p* = 0.021), the random-effects model was adopted to calculate the odds ratio (high GAS5 expression group vs. low GAS5 expression group; OR = 0.234, 95% CI: 0.153–0.358, *P* < 0.001). It demonstrated that patients with low GAS5 expression in tumor tissues were more prone to lymph node metastasis (Figure 3). In a subgroup analysis of cancer sites, we found the similar outcomes in CRC (OR = 0.353, 95% CI: 0.151–0.831, *P* = 0.017). OR for the subgroup of other cancers was 0.115 (95% CI: 0.06–0.221, *P* < 0.001). But the expression of GAS5 in NSCLC tumor tissues might not be a direct evidence of LNM (OR = 0.516, 95% CI: 0.229–1.164, *P* = 0.111). We didn't perform subgroup analyses for UC, BRC, HCC, EOC, GC, CEC, and HNSCC, there is only one paper investigating these associations between GAS5 and LNM (Figure 3) in each cancer type.

#### Association between GAS5 and HTS

**Figure 4:**
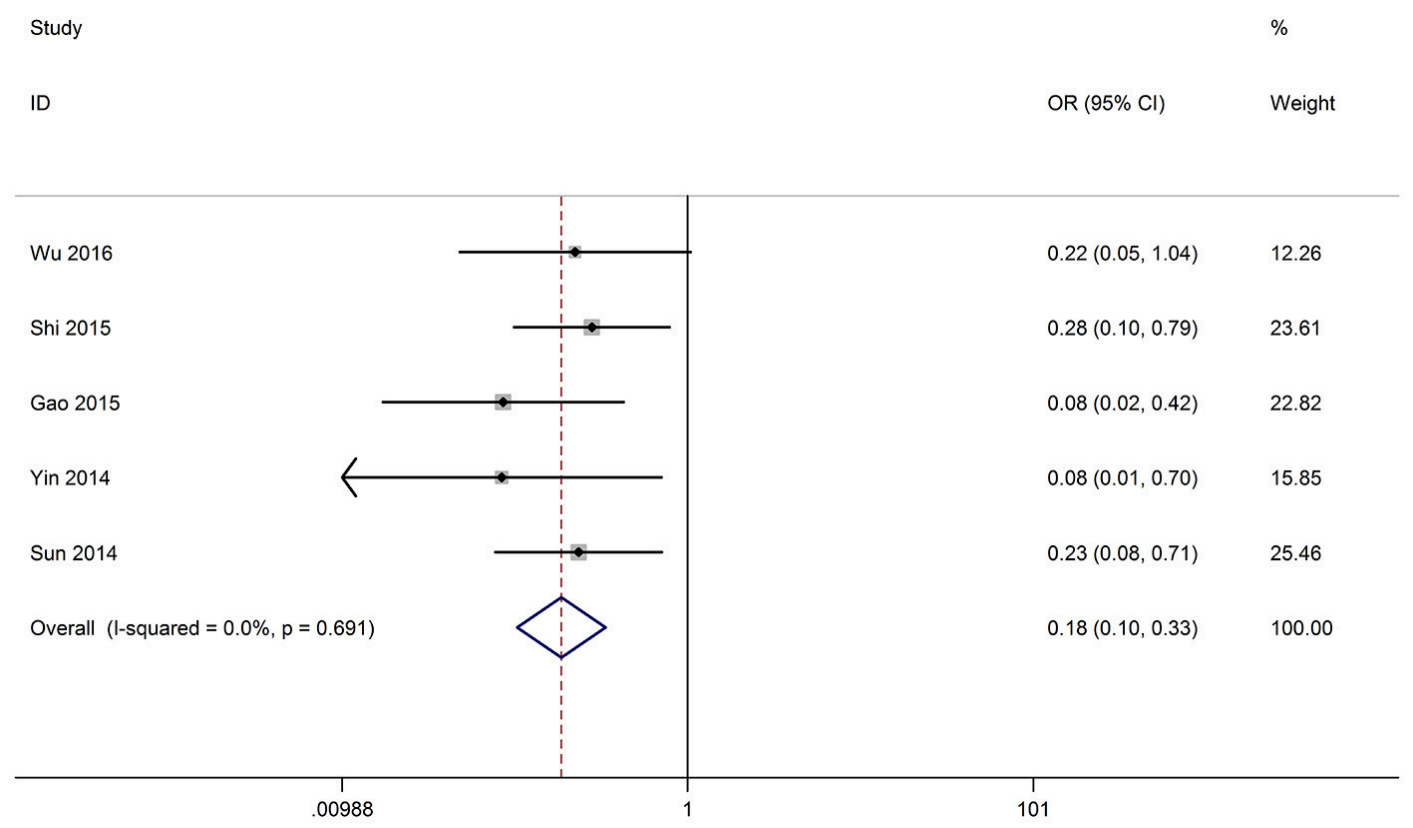
Forest plot for the correlation between GAS5 expression levels and HTS in different cancer patients.

Five studies reported the HTS of 335 patients based on variousGAS5 expression levels. The fixed-effect model was adopted because there was no heterogeneity (I^2^ = 0.0%, *p* = 0.691). The odds ratio, expressed as high GAS5 expression group vs. low GAS5 expression group, was 0.185 (95% CI: 0.102–0.333, *P* < 0.001). The result showed that patients with low GAS5 expression in cancerous tissues were more prone to high tumor stage (Figure 4). All the results were listed in the Table 3.

**Table 3:** Results of this meta-analysis.

**Outcome**	**No. of studies**	**No. of patients**	**HR/OR(95%CI)**	***P***	**Heterogeneity**	
					**I^2^ (%)**	***p*-value**
OS	10	730	1.955(1.551–2.465)	<0.001	0.0	0.728
HCC	3	153	1.893(1.103–3.249)	0.021	0.0	0.902
UC	2	188	1.653(1.185–2.306)	0.003	18.4	0.268
Others	5	389	2.641(1.625–4.204)	<0.001	0.0	0.730
LNM	7	443	0.234(0.153–0.358)	<0.001	59.6	0.021
CRC	2	90	0.353(0.151–0.831)	0.017	36.1	0.211
NSCLC	2	120	0.516(0.229–1.164)	0.111	61.6	0.107
Others	3	233	0.115(0.06–0.221)	<0.001	0.0	0.516
HTS	5	335	0.185(0.102–0.333)	<0.001	0.0	0.691

*OS, overall survival; LNM, lymph node metastasis; HTS, high tumor stage (II/III/IV); HCC, hepatocellular carcinoma; UC, urothelial carcinoma; CRC, colorectalcancer; NSCLC, non-small cell lung cancer; others, other cancer types; HR, hazard ratios; OR, odds ratios; No, number; CI, confidence interval*.

#### Sensitivity analysis and publication bias

**Figure 5:**
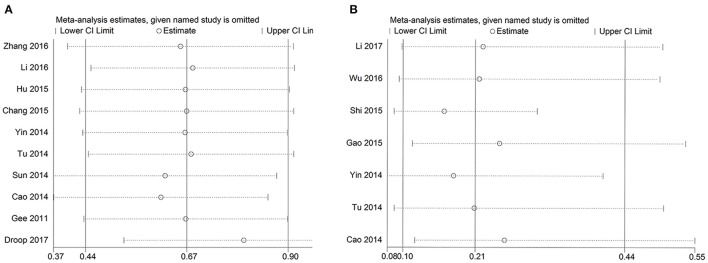
Sensitivity analysis of OS and LNM. **(A)** Sensitivity analysis of effect of individual studies on the pooled HRs for GAS5 and overall survival of patients. **(B)** Sensitivity analysis of effect of individual studies on ORs for GAS5 and lymph node metastasis of patients.

**Figure 6:**
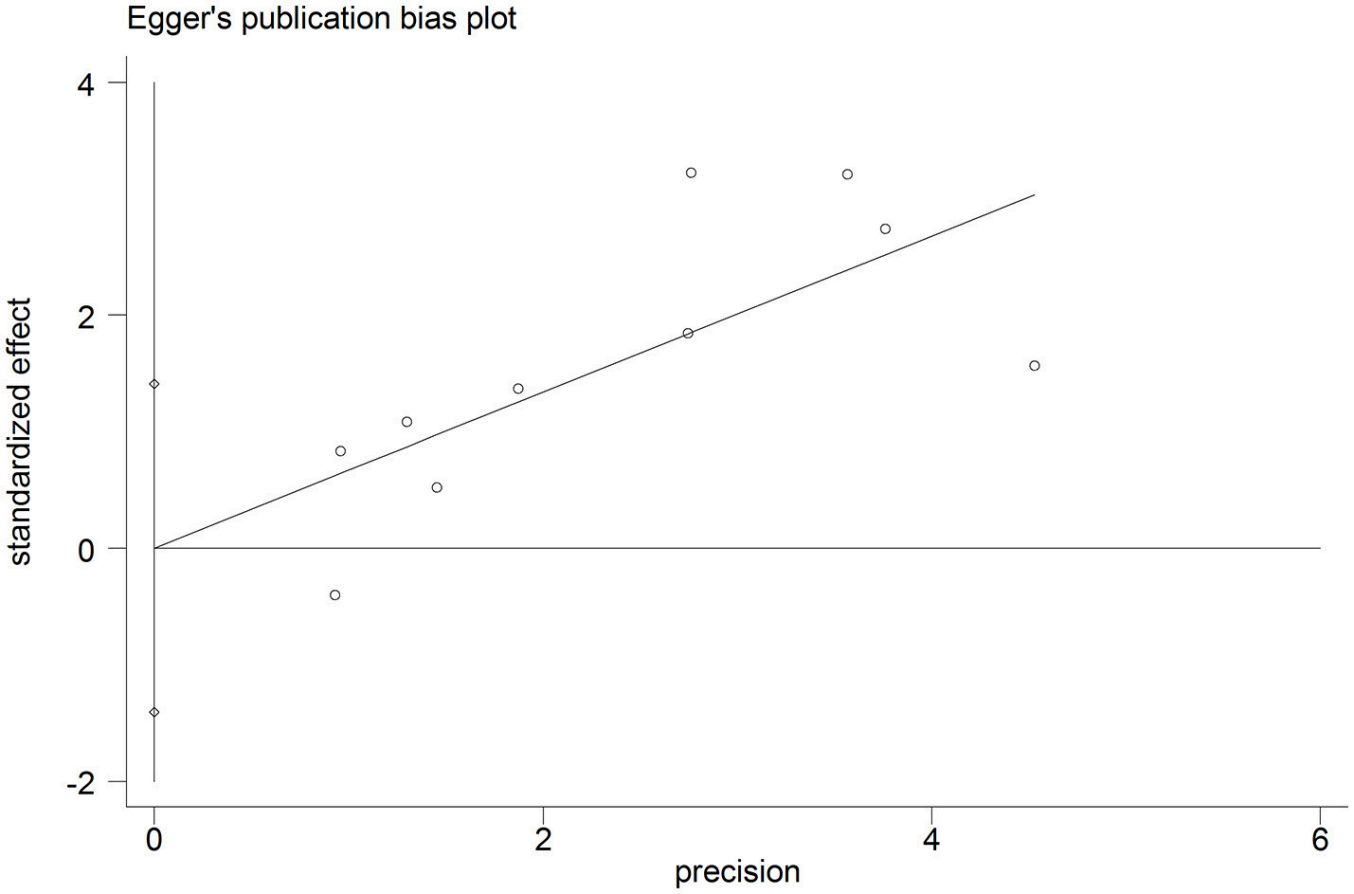
Funnel plot analysis of potential publication bias in OS group (Eegg's test): OS group.

To test the stability of the results of GAS5 and OS, we performed sensitivity analyses by sequentially removing each eligible study and the result was not significantly affected (Figure 5). We also performed a sensitivity analysis of lymph node metastasis and GAS5, and got similar results (Figure 5). We used Eegg's test to evaluate potential publication biases of the GAS5 and OS, and the result did not display obvious publication bias for the HR evaluations of OS (*p* = 0.996) (Figure 6).

## Discussion

GAS5 has been reported to be down-regulated in multiple cancers, leading to changes in tumor cell production, proliferation, apoptosis, metastasis, and survival time (Chang et al., 2016; Hu et al., 2016; Zhang et al., 2017). In our meta-analysis, we explored the relationship between the level of GAS5 expression and cancer prognostic parameters. The results demonstrated that low expression levels of GAS5 predicted poor OS in various cancers and patients with low GAS5 expression in tumor tissues were more prone to lymph node metastasis. Meanwhile, we found patients with low GAS5 expression in cancerous tissues were more prone to high tumor stage. Our results showed that low expression levels of GAS5 could be a molecular biomarker of poor prognosis in cancer patients.

As shown in Figure 2, GAS5 and OS are positively related in OS analysis without heterogeneity and publication bias: the low expression of GAS5 predicts poorer survival outcomes. To investigate whether the above analysis was applicable in separate cancers, we made a subgroup analysis. The results was HCC (HR = 1.893, 95% CI: 1.103–3.249, *P* = 0.021, P(H) = 0.902), UC (HR = 1.653, 95% CI: 1.185–2.306, *P* = 0.003, P(H) = 0.268) which meant that the above conclusions applied equally in HCC and UC. Meanwhile, we investigated the association between the GAS5 expression levels and LNM and HTS, and we found that low GAS5 expression in cancerous tissues were more prone to LNM and HTS (Figures 3, 4). However, in LNM analysis we found that the included studies existed significantly heterogeneity. So we performed a subgroup analysis according to tumor type, and the results showed that the heterogeneity disappeared obviously in CRC(P for heterogeneity = 0.211, I^2^ = 36.1%, random-effects model), and other types of cancer(P for heterogeneity = 0.516, I^2^ = 0.0%, random-effects model), while the heterogeneity still existed in NSCLC(P for heterogeneity = 0.107, I^2^ = 61.6%, random-effects model) which might be caused by the different cut-off value methods which were adopted to define the high GAS5 expression group or low GAS5 expression group. In conclusion, all these results provided strong evidence for GAS5 as a potential biomarker for the prognosis of various cancers.

Nowadays, many lncRNAs have been found to be abnormally expressed in cancer. Therefore, many meta-analysis articles, like our study, have been used to reveal the correlation of lncRNAs and cancer prognosis. Several lines of studies, meanwhile, have revealed that a lot of lncRNAs play a important role in cancer prognosis, such as TUG1, SPRY4, MALAT1 (Wang et al., 2015, 2017; Yu et al., 2017). For instance, Wang et al. found that SPRY4 is remarkably upregulated in various cancer. Thus, they performed the meta-analysis to examine the association between the SPRY4-IT1 expression level and prognosis in cancer patients. Finally, they suggested the prognostic role of SPRY4-IT1 in human cancers, and increased SPRY4-IT1 expression was closely associated with advanced features of human cancers (Wang et al., 2017). Likewise, NEAT1, as a novel lncRNA, has been recently found to be up-regulated in several cancers, contributing to tumor proliferation, apoptosis, metastasis and survival. Chen et al. conduct a meta-analysis to clarify the association between high NEAT1 expression and poor prognosis. Eventually, they concluded that NEAT1 may serve as a molecular marker and a prognostic factor for patients with various cancers (Chen et al., 2017). Additionally, among these studies, it can be found that different lncRNA has specific signaling pathways in cancers. They move the extracellular signaling molecules into the cell and then, in some way, further affect cell phenotypic changes, such as cell metabolism, proliferation, invasion, apoptosis, and so on (Wang et al., 2015, 2017; Yu et al., 2017). To further investigate the value of GAS5, we analyzed and screened the signaling pathways and mechanisms of action from all GAS5 related literatures, which will be useful for future studies on tumorigenesis (Table 4).

**Table 4:** Summary of GAS5 with their potential targets, pathways and related microRNAs entered.

**Potential targets**	**Pathways**	**Related microRNAs**	**References**
NA	Cell proliferation, invasion	miR-135b	Xue et al., 2017
NA	Cell proliferation, migration, invasion	miR-21	Hu et al., 2016; Li J. et al., 2016; Wen et al., 2017
NA	Cell proliferation, invasion and apoptosis	miR-23a	Mei et al., 2017
p53, BRCA1, GADD45A	Cell proliferation	NA	Mazar et al., 2017
P27Kip1	Cell proliferation	NA	Luo et al., 2017
IL-10, VEGF-A	NF-kappaB and Erk1/2 pathways	NA	Li et al., 2017
NA	Cell proliferation, migration and invasion	miR-137	Bian et al., 2017
mTOR	AKT/mTOR signaling pathway	miR-103	Xue et al., 2016
MT2A	NA	miR-23a	Liu et al., 2016
P53	P53 tumor suppressor pathway	NA	Shi et al., 2015; Li T. et al., 2016
D1, p21, APAF1	Cell proliferation	NA	Li J. et al., 2016
CCL1	Cell proliferation	NA	Cao et al., 2016
Bcl-2-modifying factor (bmf) and Plexin C1	cell migration, invasion	miR-222	Zhao et al., 2015
YBX1	p21 pathway	NA	Liu et al., 2015
PTEN	Cell apoptosis	miR-103	Guo et al., 2015
BAX, BAK, cleaved-caspase 3, cleaved-caspase 9	Cell proliferation, migration and invasion	NA	Gao et al., 2015
IGF-1R	EGFR pathway	NA	Dong et al., 2015
E2F1,P21	Cell proliferation	NA	Sun et al., 2014
PI3K/mTOR	Cell apoptosis, PI3K/mTOR pathway	NA	Pickard and Williams, 2014; Renganathan et al., 2014
CDK6	Cell proliferation	NA	Liu et al., 2013

*NA, not available*.

There are several limitations in our study that should be acknowledged. Firstly, the present study used the summary data rather than a specific patient data. Secondly, the methods for distinguishing the cut-off value of GAS5 in high and low expression groups were inconsonant which inevitably could cause heterogeneity. Thirdly, most of the HR values were not directly reported in these included studies. We extracted and calculated them according to the survival curves, so inevitably there might be errors. Fourthly, different treatment methods for different types of cancer patients after surgery might have great influence on the survival time, which leaded to the heterogeneity of the researches. Fifthly, we only included English related literatures that could not be so comprehensive. Sixthly, most of the studies were from China, so the conclusion might not necessarily apply in other areas. Seventhly, we only included related studies reporting OS, LNM and HTS, and the articles on other prognostic indicators were thus excluded. In the light of the above deficiencies, a more comprehensive study covering larger samples, more regions, and more indicators will be needed to confirm our results.

In conclusion, our meta-analysis found that lncRNA GAS5 could sever as a molecular biomarker to predict the prognosis of various cancers and the low GAS5 expression could indicate the poor prognosis.

## Author contributions

QG and HX performed Data extraction, HZ and JL did the data analysis. YL and WH designed the project and QG wrote the paper. YL supervised the project. WH provided financial support for the project.

### Conflict of interest statement

The authors declare that the research was conducted in the absence of any commercial or financial relationships that could be construed as a potential conflict of interest.
